# An Embodied Conversational Agent in an eHealth Self-management Intervention for Chronic Obstructive Pulmonary Disease and Chronic Heart Failure: Exploratory Study in a Real-life Setting

**DOI:** 10.2196/24110

**Published:** 2021-11-04

**Authors:** Silke ter Stal, Joanne Sloots, Aniel Ramlal, Harm op den Akker, Anke Lenferink, Monique Tabak

**Affiliations:** 1 eHealth group Roessingh Research and Development Enschede Netherlands; 2 Biomedical Systems and Signals Group Faculty of Electrical Engineering, Mathematics and Computer Science University of Twente Enschede Netherlands; 3 Department of Pulmonary Medicine Medisch Spectrum Twente Enschede Netherlands; 4 Department of Health Technology and Services Research Faculty of Behavioural, Management and Social Sciences Technical Medical Centre, University of Twente Enschede Netherlands

**Keywords:** embodied conversational agent, eHealth, self-management, design, daily life evaluation

## Abstract

**Background:**

Embodied conversational agents (ECAs) have the potential to stimulate actual use of eHealth apps. An ECA’s design influences the user’s perception during short interactions, but daily life evaluations of ECAs in health care are scarce.

**Objective:**

This is an exploratory, long-term study on the design of ECAs for eHealth. The study investigates how patients perceive the design of the ECA over time with regard to the ECA’s characteristics (friendliness, trustworthiness, involvement, expertise, and authority), small talk interaction, and likeliness of following the agent’s advice.

**Methods:**

We developed an ECA within an eHealth self-management intervention for patients with both chronic obstructive pulmonary disease (COPD) and chronic heart failure (CHF), which we offered for 4 months. Patients rated 5 agent characteristics and likeliness of following the agent’s advice before use and after 3 and 9 weeks of use. The amount of patients’ small talk interaction was assessed by log data. Lastly, individual semistructured interviews were used to triangulate results.

**Results:**

Eleven patients (7 male and 4 female) with COPD and CHF participated (median age 70 years). Patients’ perceptions of the agent characteristics did not change over time (*P*>.05 for all characteristics) and only 1 participant finished all small talk dialogues. After 3 weeks of use, the patients were less likely to follow the agent’s advice (*P*=.01). The agent’s messages were perceived as nonpersonalized and the feedback as inappropriate, affecting the agent’s perceived reliability.

**Conclusions:**

This exploratory study provides first insights into ECA design for eHealth. The first impression of an ECA’s design seems to remain during long-term use. To investigate future added value of ECAs in eHealth, perceived reliability should be improved by managing users’ expectations of the ECA’s capabilities and creating ECA designs fitting individual needs.

**Trial Registration:**

Netherlands Trial Register NL6480; https://www.trialregister.nl/trial/6480

## Introduction

The number of people having a chronic disease, such as diabetes, cancer or chronic obstructive pulmonary disease (COPD), is increasing [1]. COPD is a chronic lung disease that is progressive, and often accompanied by comorbidities, such as chronic heart failure (CHF), that further increase the risk of COPD exacerbations, hospitalizations, mortality, and costs [2,3]. Research shows that paper versions of exacerbation action plans tailored for COPD and comorbidities, embedded in a multifaceted self-management intervention, reduce the duration of COPD exacerbations and the risk of respiratory-related hospitalizations [4].

To further facilitate this chronic disease self-management in daily life, eHealth apps can be used. eHealth apps can provide patients insight into their behavior and disease by symptom monitoring, and patient-tailored and accessible support in their home setting, supervised by their health care professional at a distance [5]. Although such apps seem promising, many eHealth apps face the problem of their actual use decreasing after several weeks by a lack of user engagement [6-8]. Research indicates that a patient’s use of eHealth apps is influenced by extrinsic motivation cues, such as stimulation by care professionals and fellow patients [5,9,10]. The majority of existing eHealth apps provide such support in the form of plain text or via a text-based question–answer module, whereas face-to-face interaction remains one of the best ways to communicate health information [11,12].

A different way of providing (motivational) support includes the use of embodied conversational agents (ECAs). ECAs are defined as more or less autonomous and intelligent software entities with an embodiment used to communicate with the user [13]. By face-to-face interaction with the user, ECAs can build trust and rapport, that is, agreement or sympathy between people or groups [14]. By building trust and rapport, they could create a companionship with the user, leading to long-term and continuous use [15] and, thereby, stimulate the actual use of the underlying eHealth app. Just as a human’s appearance affects how we evaluate a human, an ECA’s appearance affects how we evaluate an ECA. When we interact with another human, or ECA, for the first time, we immediately form initial ideas about the other [16,17]. Furthermore, when we have a positive impression about another human, we tend to interact more with that human. This likely applies to human–agent interaction as well, such that we interact more with ECAs of which we have a positive first impression [16,17].

Thus, ECAs have the potential to promote engagement with eHealth apps. However, a recent review on the design of ECAs for eHealth [18] shows no clear consensus on the design of ECAs for eHealth. More specifically, the review states that emotion and empathic behavior seem to positively affect the user’s perception of the agent’s characteristics, but that these design features do not necessarily lead to users’ behavior change. The review also shows that studies mainly focus on the effect of the ECA design at first glance or after short interaction. But, to gain insight into the possible added value of ECAs in eHealth, it is important to evaluate how the ECAs should be designed for the intended context of long-term use in daily life. Only one study reports on the design of an ECA for eHealth in such a long-term, daily life setting [19]. In this study, a virtual hospital discharge nurse discussed the patient’s diagnosis and postdischarge self-care with the patient once a day at his or her hospital bed. In addition, the agent instructed the patient about medication, follow-up appointments, and self-care procedures just before hospital discharge. Questionnaires filled out after the hospital discharge showed that the patient’s perceived similarity to this agent was significantly associated with the patients liking the agent and their trust in and desire to continue with the agent. In addition, perceived similarity was associated with the patient’s working alliance with the agent—which the authors define as “trust and belief in working with the agent to achieve a therapeutic outcome.”

To develop ECAs to support users in self-management of chronic diseases, such as COPD and CHF, more research is necessary on how ECA design affects users’ perceptions of an ECA in the intended context of use: a long-term, daily life setting. Research should start in early stages of development of such ECAs, as small-scale eHealth evaluation studies focusing on usability, feasibility, and end-user experience allow researchers to gain detailed information that can be used for further improvement of an eHealth app [20]. The importance of applying user-centered design (UCD; ie, designing with end users instead of for end users by involving them in all stages of the development process) is increasingly being recognized to be valuable in health care [21,22]. By involving users to participate in the early stages of development, technical flaws can be understood and overcome [6] and the technology can be developed in such a way to reach clinical value in follow-up larger-scale studies.

This is a first exploratory study on ECA design for eHealth in a long-term, daily life setting. In this study, an ECA is implemented into an eHealth self-management intervention for patients with COPD and CHF, offered for approximately 4 months. The objective of our study was to investigate how users perceive the design of the ECA over time. In particular, how they perceive the agent’s characteristics (friendliness, trustworthiness, involvement, expertise, and authority) and the agent’s small talk, and how likely they are to follow the agent’s advice.

## Methods

### Overview

This study was performed as part of the MATCH study. The aim of the MATCH study was to investigate the feasibility of an eHealth self-management intervention for patients with COPD and CHF over a 4-month period. The ECA was implemented into this eHealth self-management intervention. The MATCH study was approved by the Twente Medical Ethical Committee and registered in the Netherlands Trial register (NL6480).

### Participants

People were included for participation in the MATCH study if they (1) had a clinical diagnosis of both COPD and CHF; (2) had at least two COPD or CHF exacerbations or at least one hospitalization for COPD or CHF in the 2 years preceding study entry; (3) were at least one week after prednisolone/antibiotics/furosemide course and hospitalization and at least four weeks after rehabilitation; (4) were at least 40 years of age; (5) were able to understand and read the Dutch language; (6) were able to use a smartphone, tablet, or PC; and (7) provided written informed consent prior to participation. People were excluded from participation if they (1) had terminal cancer or were at the end stage of another serious disease, (2) had another serious lung disease, (3) expected cardiovascular intervention within 3 months, (4) were enrolled in randomized controlled trials or a trial with study medication, (5) were waiting for a heart or lung transplantation, and (6) received renal dialysis.

### The eHealth-Supported Self-Management Intervention

**Figure 1:**
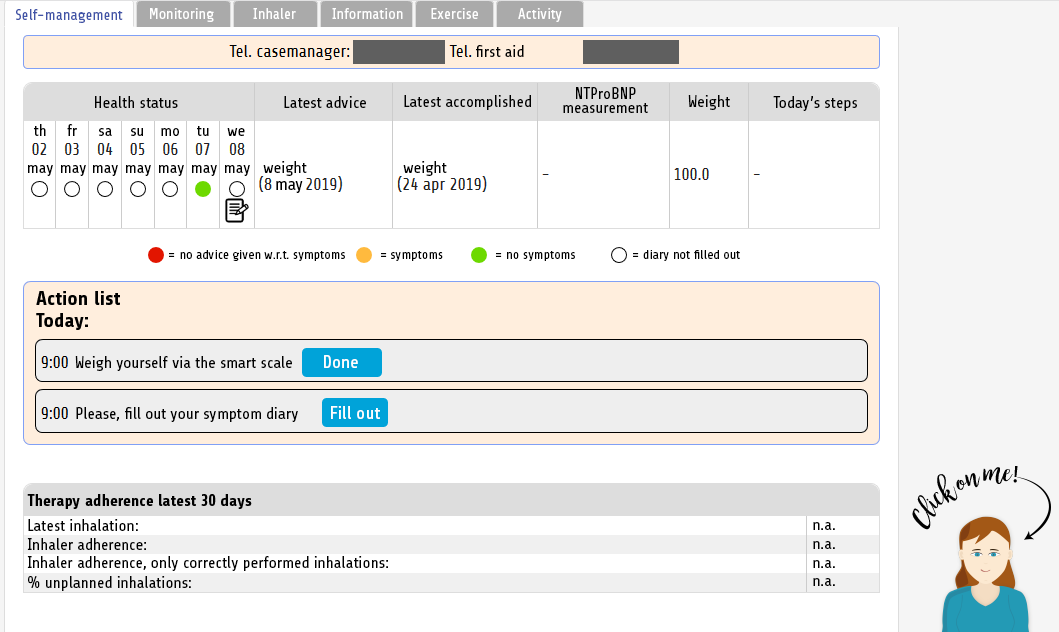
Home page of the MATCH self-management application, showing the patient’s health status and action list, and ECA. The ECA, Sylvia, was always present in the right bottom of the application. The text “click on me” and an arrow pointing to the agent were shown only before the first interaction with the agent started. ECA: Embodied conversational agent

The self-management intervention was offered through an app on a tablet (eHealth platform, Roessingh Research and Development, Enschede, the Netherlands) [23] and consisted of the modules listed in Textbox 1, as can be seen in Figure 1.

**Modules of the self-management intervention.**

**Self-management module**

- *Daily symptom diary:* registration of symptoms related to COPD (eg, dyspnea, cough), CHF (eg, weight, edema), and common comorbidities (depression, anxiety) and classification of symptoms in case of symptom deterioration determined by the patient by comparing the symptoms experienced in the last 24 hours with his or her “usual” symptoms on his or her “what are my “usual” symptoms” card. In case of any symptom deterioration, patients were asked to classify each symptom as “normal,” “slightly increased,” or “significantly increased.” The daily symptom diary was connected to a decision-support system that automatically launched self-management advice in case of worsening of the patient’s clinical condition (according to symptoms and weight). The automated decision support system was translated from an evidence-based self-management intervention including paper versions of multimorbid exacerbation action plans for patients with COPD and comorbidities [4,24].
- *Action list:* a list of actions containing (1) self-management advice determined by the automated decision-support system (eg, initiate self-treatment, perform relaxation exercises from the *exercise module*, call the case manager). In addition, the list contained (2) reminders to measure weight by a smart scale and (3) reminders to complete questionnaires.
- *Phone numbers:* to contact health care providers for support.
- *Health status:* an overview of a patient's health status during the last week, including an indication of no, slightly increased, or significantly increased symptoms.

**Monitoring module**

A detailed overview of health status, self-reported symptoms, weight, and received advice.

**Inhaler module**

Monitoring of and feedback on inhaled medication adherence and technique (add-on sensor for Ellipta Amiko Respiro).

**Information module**

Presents information about self-management including patients’ diseases and healthy behavior [4,24].

**Exercise module**

A standardized set of breathing, relaxation, and physical exercises, accompanied by videos and explanation in text.

**Activity module**

Displays daily physical activity (number of steps measured by the Fitbit Zip).

Patients were advised to use the self-management module daily by completing the daily symptom diary, monitoring their weight via the smart scale, and performing the actions on the action list. Furthermore, they were advised to use the inhaler daily. The use of the monitoring, information, exercise, and activity module was voluntarily.

### Interaction With Caregivers and Fellow Patients During Self-Management

Patients first attended 3 self-management training sessions (2 group sessions and 1 individual session with the case manager) that among other things included information regarding their diseases and training to recognize symptoms and to practice with using the self-management app. Patients started using the app after the first (group) session, so that questions regarding self-management and the technology could be answered during the next 2 sessions.

For safety during the period of app use, patients were advised to call the case manager (or general practitioner outside office hours) when symptoms did not improve after 2 days of self-treatment and when they experienced dizziness. In addition, the case manager checked health status of the patient (in the app) once per week, and called the patients when they found this was necessary. During the self-management training, patients were instructed that they could call the case manager in case of any questions or doubts. Further, regular health care (eg, visits to their pulmonary physicians and cardiologists) continued as normal during the study.

### The Embodied Conversational Agent

**Figure 2:**
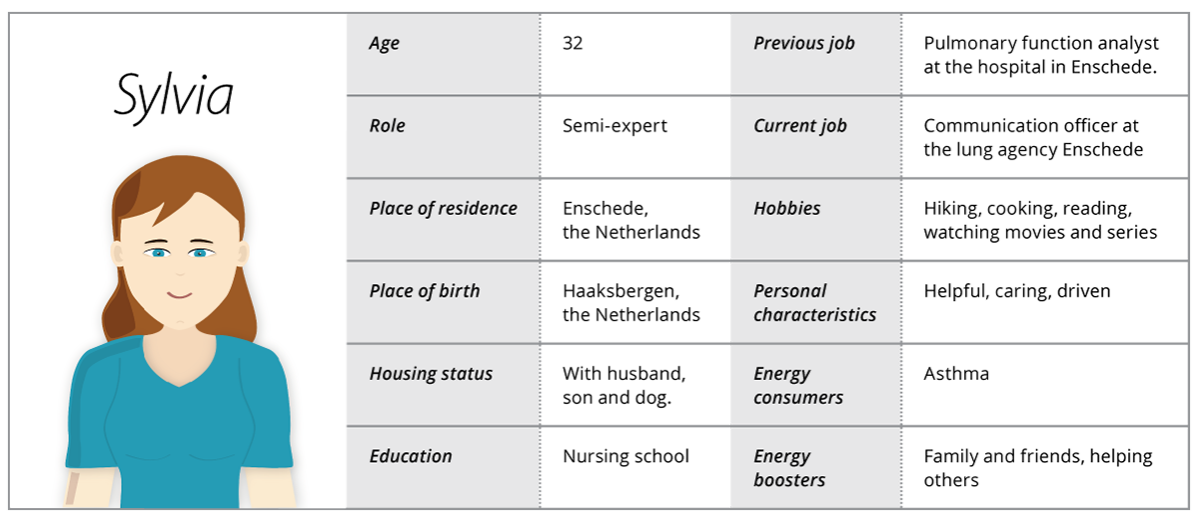
The persona of the agent Sylvia used as a guideline for writing the dialogues.

The agent characteristics found in the literature were taken into account (Textbox 2; also see Figure 2) when designing the current agent in a creative process with the developers having a description of a persona as outcome.

**Agent characteristics.**

**Gender: Female**

Research indicates that people prefer ECAs that fit their task-conform stereotypes. For health-related tasks (eg, providing medical advice) female agents are preferred [25,26], because these tasks are traditionally being undertaken by women.

**Age: Young adult**

Research indicates that people prefer young agents over older agents in the context of health, specifically in self-management for chronically ill elderly [27]. As the authors explain, a younger agent might be found more attractive.

**Cultural background: Grown up in the Twente region, the Netherlands, living in a terraced house with garden**

Research indicates people prefer agents having the same cultural background as themselves [28-30]. The cultural background of the agent is, for example, expressed in the agent’s small talk: the agent talks about activities and events related to her place of living.

In addition, to establish a full persona, additional characteristics of the persona were created. Two examples of reasoning behind the characteristics of the persona are given below. The persona used as a guideline to write the dialogues can be seen in Figure 2.

**Role: Semiexpert**

Because the self-management intervention was supported by a health care professional (nurse practitioner COPD and nurse practitioner CHF), we decided not to create a second medical expert agent. In addition, the goal of the interactive dialogues of the agent was to support patients. Therefore, we gave the agent the role of a “semiexpert,” an agent with some experience in chronic diseases (reflected in her career), but that does not act as a doctor or nurse practitioner.

**Energy consumers: Asthma**

To trigger users to identify with the agent, we decided that the agent has a chronic lung disease as well. However, to ensure the stories of the agent would not become too negative, focusing on limitations related to the disease, we decided the agent has a mild form of asthma.

The ECA, Sylvia, was implemented into the app via the use of a scalable vector graphics object, including HTML animations. The agent blinked her eyes every 10 seconds, and moved her mouth a fixed period after a new sentence appears on the screen (as if she was talking to the user). The ECA was always present in the right bottom on the pages of the self-management app. Before the user interacted with the agent for the first time, the text “click on me” and an arrow pointing to the agent were shown (Figure 1). After the first interaction, this message disappeared. When hovering over the agent, the agent increased in size.

**Figure 3:**
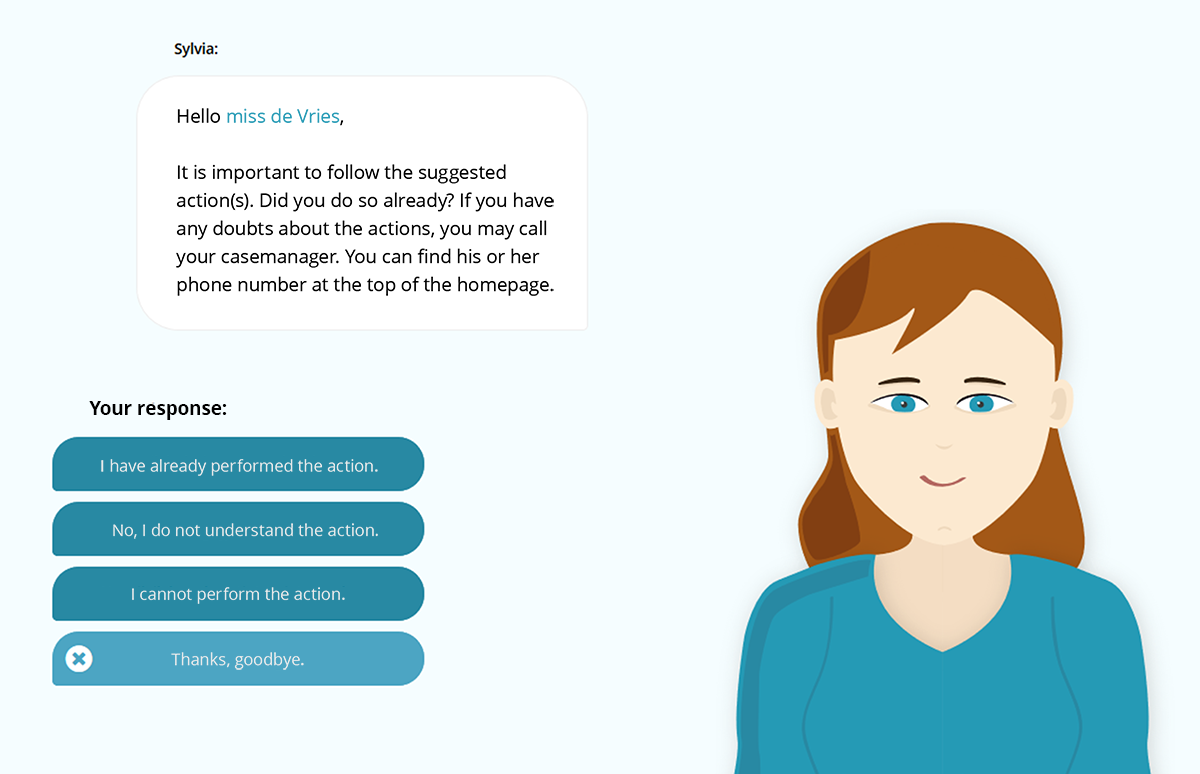
Example of a dialogue in which the agent Sylvia reminds the user to perform an action.

**Figure 4:**
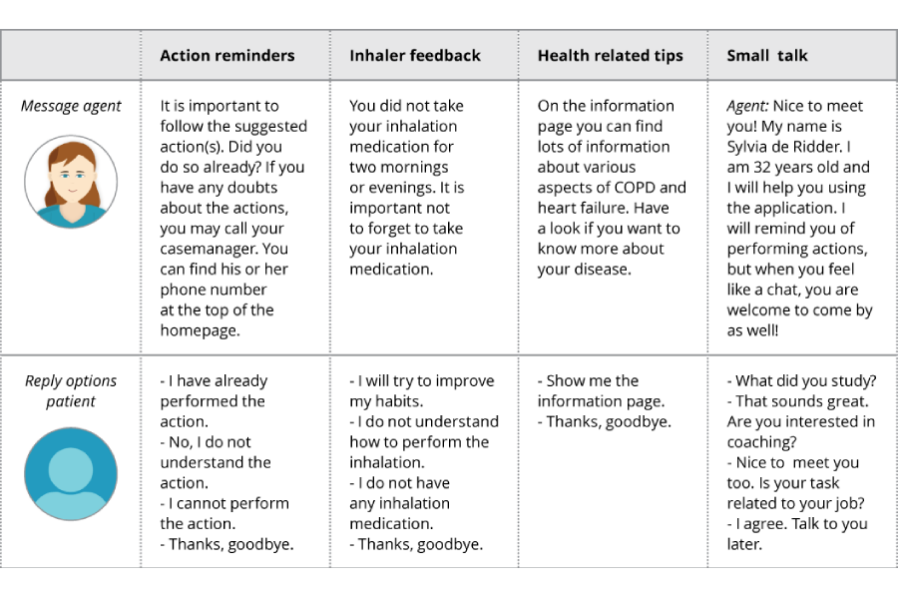
An example dialogue step for every dialogue type that could be triggered.

The content and trigger times of the dialogues were created in collaboration with experts on COPD and CHF to ensure that it was in line with patients’ daily practice. Four types of dialogues could be triggered (Textbox 3; also see Figures 3 and 4).

**Types of dialogues.**

**Action reminders**

Dialogues in which the agent reminded the patient of performing actions on the action list of the self-management app (eg, completing the daily symptom diary, weekly questionnaire, or monthly motivation questionnaire; weighing themselves; initiating medication for self-treatment of worsening symptoms; and calling the case manager for support). The agent provided the patient with a general message stating that there were uncompleted actions on the action list, but did not provide the patient with the actual content of these actions.

**Inhaler feedback**

Dialogues in which the agent informed the patient about (1) the synchronization of the smart inhaler and (2) the inhalation adherence and technique. More specifically, the first type of dialogue informed the user when the smart inhaler had not synced for either 24 or 72 hours. The second type of dialogue informed the user when the inhalation had been skipped for over 2 days, an extra dose had been taken during the last 7 days, the inhalation time of the last inhalation deviated too much from the average duration of the inspiratory flow, and when the position of the device was not optimal.

**Health-related tips**

Dialogues in which the agent provided the patient with several health-related tips, such as accessing information sources or small actions to perform in daily life. Some of the tips referred to information provided at pages in the self-management app.

**Small talk**

Chitchat dialogues to increase the patient’s engagement [31], stimulating the use of the underlying app. The small talk dialogues were designed as a daily soap series to trigger the patient’s curiosity about the continuation of the story. The small talk was split up into 7 “episodes,” all containing multiple dialogue steps around a certain theme (the introduction and Sylvia’s housing status, husband, child, neighbor, hobbies, and dog). When the patient finished an episode of the small talk, the next episode was unlocked the next day. In the meantime, when the patient clicked on the agent, the agent informed the patient that she does not have time to talk until tomorrow (ie, showed a “wait till tomorrow” message). When the patient finished all 7 episodes, the agent told the patient that she had nothing more to say.

Small talk dialogues could be triggered by the user by clicking on the agent on the home page of the self-management app. The other dialogues were triggered by the system at predefined trigger times:

- *Action reminders:* 1, 2, and 3 hours after an action was added to the action list and not yet performed;
- *Inhaler feedback:* when incorrect inhaler use was measured;
- *Health-related tips:* each day at 15:00 pm;
- *Small talk:* each day at 14:00 pm (only when the patient did not yet initiate a small talk dialogue that day by himself or herself and the small talk was not yet finished).

Each dialogue consisted of one or multiple dialogue steps, containing one or multiple answer possibilities for the user. The agent message was displayed in text and not communicated via speech. An example of the interface of the dialogue step can be seen in Figure 3. Examples of the content of the dialogue steps for every dialogue type can be seen in Figure 4.

### Procedure

**Figure 5:**
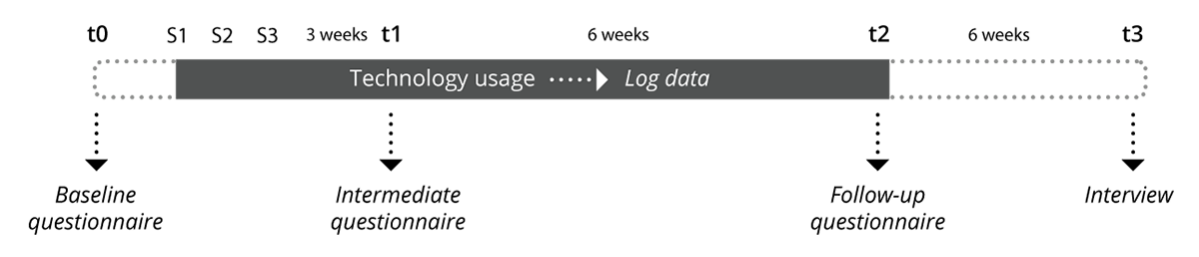
The procedure of the study. S1 = first group session, S2 = individual session, S3 = second group session, t0 = start, t1 = 3 weeks from the start, t2 = 9 weeks from the start, t3 = 15 weeks from the start.

Figure 5 provides an overview of the study procedure. Written informed consent from the participants was obtained prior to study participation. Then, the participants filled out the baseline questionnaire at home (t0). At this point, the participants had not yet seen the app and were not aware of the existence of an agent in the app. The participants were introduced to the agent for the first time in the baseline questionnaire, as a picture of the agent was attached to the questions regarding the agent. The agent was introduced as a hypothetical coach. During the first group session (S1) participants received a tablet, step counter (Fitbit Zip), and smart scale to be used with the app. After this meeting, the participants could already start using the app and sensors were provided. In a second, individual meeting, patients practiced with using the eHealth app according to their individual symptoms (S2). In the second group session (S3), some last questions with respect to self-management and the technology were answered. After the second group session, participants received the add-on sensor for the inhaler and afterward all patients used the app and sensors. Two weeks after the last group session (S3), participants received the intermediate questionnaire (t1). After 9 weeks of use, users received the follow-up questionnaire (t2). Technology usage was logged during the complete period of use. After the end of the use period, the participants were interviewed by an independent interviewer (AR) (t3).

### Design and Measurements

**Figure 6:**
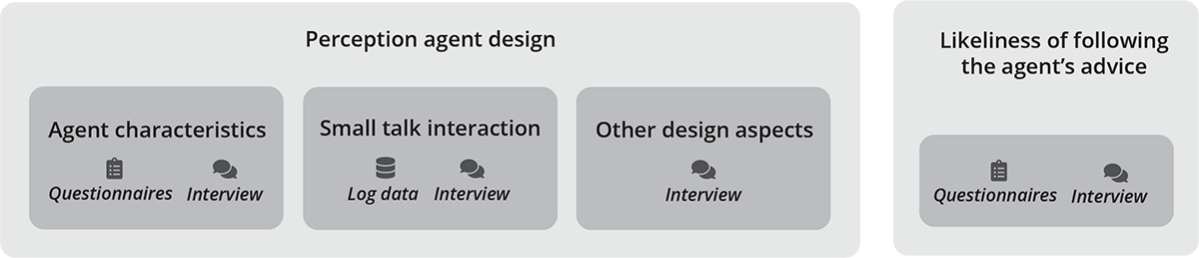
Study measurements to evaluate the user’s perception of the agent design and the user’s likeliness of following the agent’s advice.

We used a mixed-method design, combining both quantitative and qualitative research methods: questionnaires, log data, and semistructured interviews (Figure 6).

The patient’s perception of the characteristics of the agent and likeliness of following the agent’s advice were measured via (1) a baseline questionnaire at t0, (2) an intermediate questionnaire at t1, and (3) a follow-up questionnaire at t2. These paper self-reported questionnaires assessed the patient’s perception of:

- Five characteristics of Sylvia (the agent in the MATCH self-management app): friendliness, trustworthiness, involvement, expertise, and authority.
- The importance of these 5 characteristics of an ECA for self-management in general.
- The likeliness of following Sylvia’s agent’s advice.

The questions on users’ perceptions of these characteristics and likeliness of following an ECA’s advice were similar to those of 2 other studies [32,33]. All items were assessed by ratings on a 7-point Likert scale. In addition, the baseline questionnaire contained questions related to the patient’s characteristics.

Furthermore, small talk interaction was analyzed using (4) log data. The dialogue history of the small talk of the ECA with the patient was logged on the server. For each patient, the date and time of dialogues triggered by either the system or the user were logged. Furthermore, the patient’s selected responses were logged per dialogue step of a triggered dialogue, including a date and time.

Lastly, the patient’s impression of the agent’s characteristics, the likeliness of following the agent’s advice, the small talk, and other design aspects were gathered in (5) semistructured interviews.

### Data Analyses

Statistical analyses were performed in SPSS 25 (IBM). The respondents’ age was treated as a continuous variable, whereas all other respondents’ characteristics were treated as categorical variables and responses on Likert scale questions as discrete (ordinal) variables. In the questionnaires, the 5 agent characteristics were classified as low (a score from 1 until 3), neutral (a score from 3 until 6), or high (a score from 6 until and including 7) on applicability to Sylvia and important characteristic for an ECA for self-management in general. The same classification was used for the user’s likeliness of following advice.

For all relations, a related-samples Friedman 2-way analysis of variance by rank was performed as appropriate. The Holm–Bonferroni method was used to correct for multiple comparisons: the comparisons of the ratings for the characteristics of Sylvia and ECA for self-management in general and the likeliness of following Sylvia’s advice at t0 (before use), t1 (after 3 weeks of use), and t2 (after 9 weeks of use).

The interviews were transcribed by the interviewer (AR) using automatic transcription in Amberscript and a manual check afterward. Another researcher extracted the interview data focusing on the MATCH agent or ECAs in general (StS). Then, the remaining interview data were thematically analyzed by 2 researchers independently (StS and MT). All themes were grouped either under (1) the patients’ perceptions of agent characteristics, (2) small talk interaction, or (3) other design aspects. The themes were coded retrospectively using ATLAS.ti 8, based on the steps proposed in [34]: one researcher (StS) created a first coding scheme and labeled all the data accordingly. A second researcher (MT) used the coding scheme to code a subset of the data. Disagreements between the first and second researcher were discussed and overcome, leading to an updated coding scheme. The first researcher used that updated coding scheme to re-code all data entries and the second researcher then independently re-coded a new subset. Again, disagreements between the 2 researchers were discussed and overcome, leading to the final coding scheme used by the first researcher to re-code all data one final time.

## Results

### Baseline Demographics

Eleven patients (7 male and 4 female) completed the study procedure until t2, of which 9 agreed to participate in the interview at t3. The age of the participants (n=11) ranged from 49 to 83 years (median 70 years). The highest educational degree for the majority of the participants was high school or vocational education; 1 participant had a university degree. Three participants lived alone, while the others lived with their partner. Four participants indicated that their partner is their informal caregiver, whereas the others said they do not have an informal caregiver. Self-reported tablet skills were high for 4 participants, 3 did not have any experience with a tablet yet, and the rest had some experience.

### Patients’ Perceptions of Agent Characteristics

Table 1 shows the patients’ perceptions of the characteristics of Sylvia and of the important characteristics for an ECA for self-management in general over time. At t0, t1, and t2 Sylvia was rated high on friendliness; on t1 Sylvia was rated high on reliability and low on authority. For all other characteristics, the median rating of the agent was neutral at t0, t1, and t2. In addition, at each point in time, the agent characteristic *authority* was rated neutral on important characteristic for an ECA for self-management. Expertise, reliability, and involvement were rated high on important characteristic for an ECA for self-management in general. Friendliness was rated high on importance at t0 and t2, and neutral on importance at t1.

**Table 1:** Comparison of the patients’ ratings of Sylvia’s characteristics and the patients’ ratings of the important characteristics for an ECA for self-management in general at t0 (before use), t1 (after 3 weeks of use), and t2 (after 9 weeks of use) using a Friedman 2-way analysis of variance by rank.

Ratings		n	t0, median (IQR)	t1, median (IQR)	t2, median (IQR)	*P* value
**Sylvia’s characteristics**						
	Friendliness	9	6.0 (4.0-7.0)	6.0 (4.5-6.0)	6.0 (4.0-7.0)	.45
	Expertise	8	5.0 (4.0-7.0)	5.0 (4.0-6.0)	5.0 (4.0-7.0)	.47
	Reliability	9	4.0 (4.0-7.0)	6.0 (3.5-6.0)	4.0 (3.8-7.0)	.77
	Authority	8	4.0 (2.3-4.8)	2.0 (2.0-5.5)	4.0 (2.3-5.5)	.64
	Involvement	8	5.5 (4.0-7.0)	5.0 (4.0-6.0)	4.5 (3.3-7.0)	.68
**Important characteristics for an ECA for self-management**						
	Friendliness	9	6.0 (4.0-7.0)	5.0 (4.0-6.0)	6.0 (4.0-7.0)	.43
	Expertise	9	7.0 (7.0-7.0)	7.0 (6.0-7.0)	7.0 (6.5-7.0)	.25
	Reliability	9	7.0 (6.5-7.0)	7.0 (6.0-7.0)	7.0 (6.5-7.0)	.84
	Authority	9	4.0 (2.0-6.0)	4.0 (1.5-5.5)	4.0 (3.0-4.5)	.65
	Involvement	9	7.0 (6.0-7.0)	6.0 (6.0-7.0)	6.0 (4.0-7.0)	.78

In the interviews, the patients commented on some of the above measured characteristics. We identified the themes *friendliness, reliability, expertise,* and *authority*. One participant found the agent (Sylvia) *friendly*, whereas another did not indicate whether Sylvia was friendly, but stressed that an agent for self-management support should be friendly. Furthermore, Sylvia was not always seen as *reliable*, supported by a participant indicating that the messages of Sylvia were based on data from a nonreliable Fitbit. Although Sylvia did not provide advice based on the Fitbit data, this participant might have thought this was the case. Another participant indicated that Sylvia sometimes gave tips that did not fit the participant’s individual situation; for example, suggesting to perform physical activity when having filled out symptoms in the diary, affecting the agent’s reliability. One participant especially commented on the agent’s *expertise*, calling Sylvia “a stupid woman.” In addition, one participant commented on *authority*, saying

She could be your girl next door...If I have medical complaints, I prefer an authority to explain what to do or not to do.

### Small Talk Interaction

**Figure 7:**
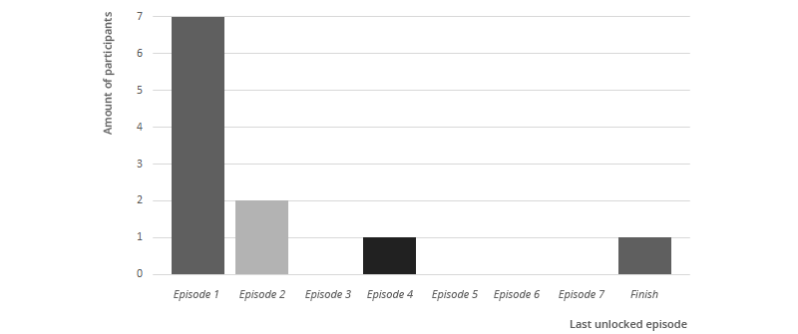
The number of study participants that unlocked a particular small talk episode.

Figure 7 shows how many participants unlocked particular episodes, based on the log data. Seven out of the 11 participants did not finish the first episode. Two participants finished the first episode and, therefore, unlocked episode 2. In addition, 1 participant unlocked the episodes until 4. Finally, 1 participant finished all 7 small talk episodes. Two participants were shown a “wait till tomorrow” message, as they already finished a small talk episode that day; one participant saw the message 3 times; and the other 5 times. Finally, the participant that finished all dialogues was shown the message that the small talk was finished for 45 times, meaning this participant clicked on the agent to receive a new small talk dialogue for 45 times, whereas the dialogues were finished.

In the interviews, participants had a few comments on the agent’s small talk. One person did not notice that Sylvia talked about her own life. Five participants said they were not interested in the small talk. However, 2 of them thought that people that feel lonely might be interested. One participant (not the participant that finished all the small talk dialogues) showed a more positive attitude toward the agent’s small talk:

Sylvia could talk nicely, she told me many things, for example, that she was lonely.

### Other Design Aspects

In addition to the agent characteristics and small talk, the analysis of the interviews resulted in the following themes with respect to the agent design: *the agent’s appearance*, *frequencies of the messages*, *timing of the messages*, and *the interface design*.

First, *the agent’s appearance* was evaluated. One participant preferred to interact with a photo-realistic nurse, instead of a computer-animated figure, because a photo-realistic nurse would make the interaction more personal. The participant also stated:

I am not impressed by a cartoon figure.

Furthermore, the participant described the agent as

A male or female such as on the doors of bathrooms.

Another participant also preferred the agent to look like a nurse; this participant particularly commented on the agent’s clothing:

Put a white coat and a stethoscope on her.

The participant described that an agent having a white coat and stethoscope would look more authoritative than the current agent in a t-shirt. Lastly, 1 participant liked that the agent was a woman, because the participant hates listening to men.

Second, 2 participants particularly commented on the *frequency of the messages from the agent*: in their view, they received too many messages. One of them indicated that, therefore, he or she closed the dialogue before reading it.

Third, with respect to the *timing of the messages*, 1 participant would like to receive conformation messages when performing actions (real-time feedback on actions), whereas another suggested that the agent should come back to topics discussed before, as illustrated by:

But, then, ask the next day: ‘Did you read that? Did you do this?’

In addition, 2 participants indicated that Sylvia provided unwanted and unsolicited information. One argued that she started to talk about a topic, regardless of whether the user was interested in that topic at a particular moment. This participant, instead, would like to receive the information when asking for it. The other participant argued that Sylvia provided advice when the participant felt well, whereas this participant only wanted to receive advice when not feeling well. Also, 2 participants said that they did not always have the time to follow the suggested actions or tips when receiving them from the agent. One was really annoyed when receiving messages, like “think about your exercises,” straight in the morning, the other explained:

When I have to go to work, I do not have time to watch at a 15-minute video.

Finally, 2 participants found it annoying that the agent already started giving reminders, when opening the app, not having the chance to even perform the action, illustrated by:

Look, what really bothered me was that, in the morning, I turned on the device and it [the agent] started with saying: ‘Did you follow the instruction?’ Well, I did not see any instruction yet.

### Likeliness of Following the Agent’s Advice

Table 2 shows the results of the related samples Friedman 2-way analysis of variance by rank, comparing the participant’s likeliness of following the agent’s advice over time. On t0, t1, and t2 Sylvia scored neutral. A significant difference (*P*=.01) in the distribution of the values over time was found.

As a second step, pairwise comparisons of t0 and t1, t0 and t2, and t1 and t2 showed no significant difference between t0 and t2 (*P*=.07) and t1 and t2 (*P*=.48), but did show a significant difference for the pair t0–t1 (*P*=.01). The participant’s indicated likeliness of following the agent’s advice statistically dropped at t1 compared with t0.

**Table 2:** Comparison of the ratings of participants’ likeliness of following Sylvia’s advice at t0 (before use), t1 (after 3 weeks of use) and t2 (after 9 weeks of use) using a Friedman 2-way analysis of variance by rank.

Comparison	n	t0, median (IQR)	t1, median (IQR)	t2, median (IQR)	*P* value
Likeliness of following advice	9	6.0 (4.5-7.0)	3.0 (2.0-4.0)	4.0 (2.5-5.0)	.01^a^

^a^Statistical significance is considered if *P*<.05.

In the interviews, the majority of the participants indicated that they would not *follow the agent’s advice*. Two of them questioned the agent’s reliability. In line with this, another participant (male) indicated that he would first go to a doctor to verify the agent’s advice of taking prednisolone. Although it should be noted that the agent did not provide the patient with advice on taking prednisolone directly, the agent only mentioned that there was an uncompleted action on the action list, which might have been taking prednisolone. However, the actual advice was determined by the automatic decision support system. Another participant argued that the agent did not respond to user input and, therefore, did not find the agent’s advice valuable. One participant mentioned not listening to a cartoon figure, and another stated:

I do not listen to a device, I do listen to people.

Furthermore, a participant indicated that adults have their own responsibility, and therefore, this participant did not feel the need for an agent to suggest what to do. One participant argued not having the time to follow the advice, and therefore, not seeing the benefits of the agent’s advice. By contrast, 3 participants said they sometimes did follow the agent’s advice. One of these participants sometimes performed the physical exercises advised, as this participant valued the exercises. Another indicated to follow the advice of calling the case manager or reading information pages, but would not follow an advice to start prednisolone. The participant said that being wrongly advised to take prednisolone could have negative health consequences, believing that the technology’s advice is not always correct. Furthermore, a participant (female) indicated to call the case manager if advised, as she would normally also have done so. As explained before, it should be noted that the actions of calling the case manager and taking prednisolone were part of the action list of the self-management app, but were not presented by the agent itself.

### General Attitude Toward the ECA Design

The last theme we identified was *general attitude toward the ECA design*. The theme does not correspond to our main objective, but we present the findings to provide insight into the context of the results described above. The interviews show that the majority of the participants (n=7) did not think that Sylvia had any value, illustrated by comments, such as:

I do not have any connection with Sylvia.

Sylvia is not it.

Arguments supporting this opinion were the agent’s statements being too obvious, general, or simplistic: a participant described that it was clear that the dialogues were not personalized, but a result of a general set of if–else statements.

Also, Sylvia led to lots of frustration and annoyance, as supported by statements such as:

I found this female extremely annoying.

Sylvia was a very irritating woman.

Frustrations were caused by Sylvia providing incorrect feedback on the inhalations and suggesting actions not fitting the user’s health status, as illustrated by a participant:

I thought: “Gosh, what are you talking about? I’m not complaining about respiratory problems.”

One participant (male) particularly indicated he would like to switch off the agent. By contrast, the interviews showed some positive attitudes toward Sylvia. One participant said that the agent triggered laughing, as Sylvia would adapt the conversation to the answers given. This participant explained:

Occasionally, if I felt bad, I could laugh again.

This participant also said that Sylvia made the app more personal, for example, by addressing the user by his or her first name:

It [Sylvia] creates a slightly more informal atmosphere, which I always like, I feel a bit more free.

In addition, this participant believes people should get used to interacting with agents:

When you are at the station, you have this as well [...]. You enter the station and then you face a digital agent. This is something we should get used to, I think.

Lastly, participants suggested improvements for the interaction with the agent. First, 2 participants explained they would like to be able to type a question in an input field and receive a personalized answer. One of them sketched a scenario in which a patient, who is not feeling well, types in a question into an input field, for example “I am feeling stuffy, but have taken prednisolone: what should I do?” and the app would respond with an answer 24/7. It should be noted that one participant did not understand that Sylvia was a digital agent. He thought that Sylvia represented one of the real people involved in the self-management meetings.

## Discussion

### Principal Findings

This exploratory study aimed to investigate how an ECA’s design is perceived by its users when implemented in a long-term, daily life setting. Although the results of this study should be interpreted carefully, as this is a small-scale study, they provide first insights into an ECA’s design for self-management and guidelines for follow-up work in terms of both development and evaluation. Our study shows that the patient’s perception of friendliness, expertise, reliability, involvement, and authority of the ECA did not change over time. The majority of the users were not interested in the agent’s small talk and the likeliness of following advice decreased after 3 weeks of use.

First, our study shows that the perception of the agent’s characteristics at first glance was similar to that after 2 weeks and 9 weeks of use, suggesting that the user’s first impression does not change over time. To the best of our knowledge, there are no studies on how these perceptions change over time. But, ter Stal et al [32] showed that an agent’s design affects the user’s perception after short-term interaction, while Zhou et al [19] showed that this also applies to long-term interaction.

How do you design an agent for self-management that creates positive impressions that persist? Our results suggest that an agent for self-management should be friendly, reliable, and involved and should have expertise, because patients rated these characteristics as important. Cafaro et al [35] found that an agent’s friendliness was related to the user’s number of agent approaches and likeliness of future encounters with the agent. In addition, the characteristics expertise, reliability, and involvement are found to be important aspects of persuasive systems [36], and eHealth apps in particular [37-39]. However, taking this together does not provide much evidence on what agent characteristics are especially important. In our study, patients gave higher scores for Sylvia’s reliability and involvement than for Sylvia’s authority. However, patients also indicated that an agent’s authority is less important than expertise and reliability. This emphasizes the importance for future ECA design studies to ask for both the perception of the characteristics of the agent designed (ie, the scoring) and the perceived importance of these characteristics for an agent in the specific context. With respect to the agent’s authority, our study was indecisive. Different from quantitative data, qualitative data indicated that patients do prefer an agent portraying authority. These contradicting results might be caused by the patients actually meaning that the agent should have expertise, as they indicate in the interviews that “the agent should have authority regarding the topic.” Nevertheless, research confirms that people tend to prefer agents designed to fit their task [25,26]. In the context of a self-management intervention for COPD and CHF, we could increase the agent’s expertise by having the agent wear a doctor’s coat. Whether this actually results in a better perception of the agent should be further investigated.

In addition, our study showed that a photorealistic agent could result in users being more likely to follow the agent’s advice, compared with a static cartoon. Van Wissen et al [27] indicated that a more realistic agent appearance increases users’ likeliness of following the agent’s advice and leads to increased learning of students supported by a pedagogical agent [40]. This increased learning might possibly also apply to a patient’s learning about chronic disease self-management. In addition, a realistic agent appearance leads to higher user engagement [26,27,41] and a positive perception of the agent’s characteristics, such as its trustworthiness and competence [27]. By contrast, we should avoid the agent being too human-like, as then a mismatch between the users’ expectations of the agent and the agent’s actual capabilities—a so-called negative adaptation gap—could be created, resulting in the users being disappointed [42]. Future work should investigate the sweet spot between facilitating expertise (through more realism) and managing expectations of intelligence (through reduced realism).

Furthermore, our study showed that the majority of the users was not interested in the agent’s small talk. Although we expected that the small talk would increase users’ engagement through companionship building with the agent [31], this seemed not the case. A possible explanation might be that the amount of small talk might have exceeded the amount of health-related content, and, therefore, distracted the patients from the actual goal of the app: self-management. We expect that it is better to adapt the amount of small talk to the user, for example, by tracking the user’s interaction in the small talk dialogues and adapting the amount of small talk in the future accordingly (ie, users that interact in small talk more often receive small talk more frequently). In addition, the content of the small talk could be adapted to the user. Research shows that tailoring health messages toward personal characteristics pays off [43], suggesting that a user’s demographics might affect the type of small talk the user is most engaged by. Future work could focus on how small talk can be personalized to fit the users’ personal values and interests.

Lastly, our results show that patients were less likely to follow the agent’s advice over time. We expect that the participants had a negative adaptation gap, meaning that their expectations of the agent’s capabilities did not match the agent’s actual capabilities [42]. After a few weeks of use, the users might have realized that the agent’s messages did not always fit their situation, resulting in a decrease of their likeliness of following the agent’s advice. In addition, the agent design led to frustrations, mainly caused by nonpersonalized content and inappropriate feedback, affecting the agent’s reliability. Such a mismatch of the content of the agent’s message with the user’s personal situation was also found by ter Stal et al [33] who evaluated ECAs for health assessment of older adults. Personalizing the agent by providing more specific feedback on user input and health-related data (eg, sensor inhaler data) might improve the likeliness of individuals to follow the agent’s advice. However, the technology readiness level (TRL) of the ECA fits the exploratory character of the study, as explained in the staged approach of telemedicine evaluation [20]. In the first stages of an telemedicine evaluation (ie, evaluation of feasibility and user experience), exploratory studies are used to investigate and increase quality of technology, while in later stages, clinical value can be researched with more mature technology in large-scale studies [6,20]. As a consequence, participants’ expectations of the technology, especially that of the agent, might have exceeded the functionalities and quality of the technology used. Therefore, we emphasize the importance of managing the participants’ expectations of the technology used in a study; that is, they should match the actual TRL of the technology. For an agent in particular, it needs to be explained what the user can expect from the agent, which allows one to focus on the objective of the study.

Our results underpin the importance of applying UCD methods throughout the various development phases of eHealth apps [21]. By incorporating end users early in the development of an ECA for self-management, we learned whether our hypotheses about users’ perceptions of the ECA design were correct and gained new insights into how to adapt the design of the ECA to the end users in a next design iteration. The importance of such UCD is recognized more frequently in the field of human–computer interaction, reflected by the development of standards, such as the ISO 9241-210 [44-46]. As described by Mithun et al [44], the ISO 9241-210 clarifies UCD principles and describes that a design process should be iterative; the iteration is the review and refinement of design specifications. Czaja et al [47] stress the importance of UCD for products targeting older adults. They indicate that older adults have unique usability constraints compared with younger adults. As they describe, when usability is improved for older adults, it is also improved for younger adults. Therefore, they stress to take into account the context and characteristics of older adults in the design process. We did so, as many of our participants were older adults.

### Strengths and Limitations

The strength of our research is that we evaluated the perception of an agent’s design at an early development stage with the end users. Furthermore, participants interacted with an agent in a daily life setting and during a longer period of use: a setting which is rare in agent research, mostly consisting of research on short-term interaction with agents in laboratory settings. However, this long-term, daily life setting put quite some load on participants. Because of the exploratory character of the study, a limited number of patients participated, which should be taken into account when interpreting the results. However, the results can provide guidance for follow-up agent development and evaluation. Furthermore, participants used a Fitbit, smart scale, and a smart sensorized inhaler in combination with the self-management app. Many participants complained about the sensors not working properly. This might have affected the participant’s perception of the agent, as some of the messages of the agent were based on incorrect sensor information. Lastly, the interviews focused on all elements of the self-management intervention, not specifically on the design of the agent. Not all participants provided information related to the research question of this study, and, therefore, we should be careful with interpreting the results of the interviews.

### Conclusion

This exploratory study provided first insights into ECA design for long-term, daily use. An agent’s design is important for patients to establish a good first impression of the agent, which remains during long-term usage. Based on our findings we expect that ECAs do have the potential to be used for self-management, but several design aspects should be investigated in order for ECAs to become successful for increasing engagement in eHealth. When designing ECAs for self-management, we recommend designing an agent that is friendly, reliable, involved, and that has expertise, such that designers can implement and evaluate personalized content and small talk with sufficient variation, and find a good balance between small talk and health-related content. Careful consideration should be given to the apparent realism of the agent to find the sweet spot between facilitating expertise (through more realism) and managing expectations of intelligence (through reduced realism). In combination with managing the user’s expectations of the agent capabilities, a personalized ECA design fitting individual needs could increase the agent’s reliability and, therefore, the user’s likeliness of following the agent’s advice. This way, the ECA design could be upgraded to a higher TRL for which the added value and clinical benefits can be evaluated in future research.

## Abbreviations

CHF: chronic heart failure
COPD: chronic obstructive pulmonary disease
ECA: Embodied conversational agent
TRL: technology readiness level
UCD: user-centered design
